# 

**Published:** 1808-10

**Authors:** 


### NEW MEDICAL PUBLICATIONS.

A Treatise on the Operation of Lithotomy; by Robert Allan, Surgeon, folio, 1l. 11s. 6d.

Modern Medicine, containing a brief Exposition of the principal Discoveries and Doctrines that have occasioned the recent advancement of Medical Philosophy, with Strictures on the present State of Medical Practice; by David Uwins, M. D. 8vo, 6s.

---

### To CORRESPONDENTS.

We are much obliged by a Communication from Dr. BLEGBOROUGH, and shall pay due attention to it, if necessary.

